# Concocting Cholinergy

**DOI:** 10.1371/journal.pgen.1004313

**Published:** 2014-04-24

**Authors:** Artur Kania

**Affiliations:** 1 Institut de Recherches Cliniques de Montréal (IRCM), Montréal, Quebec, Canada; 2 Departments of Anatomy and Cell Biology, and Biology, Division of Experimental Medicine, McGill University, Montréal, Quebec, Canada; 3 Faculté de Médecine, Université de Montréal, Montréal, Quebec, Canada; University of California Los Angeles, United States of America

The neuronal diversity in our brains is staggering. Understandably, uncovering the molecular rules that govern it is a very difficult pursuit. Its starting point should most certainly be a *catalogue raisonné* of this diversity, ordered perhaps according to the relatively few neurotransmitters employed in the nervous system. Historically, this has been achieved in descriptive studies; first using histochemical approaches and later antibody staining for the neurotransmitters, their synthetic enzymes, and the transporters that load them into secretory vesicles as well as for their receptors on target cells. How the expression of such neurotransmitter machinery is coordinated to endow a neuron with a “neurotransmitter identity” (NtI) is a big piece of the molecular puzzle of neuronal diversity. Cho et al. provide a glimpse into this question by uncovering how an acetylcholine NtI gene transcription program is established in two very different vertebrate neuronal populations [1].

Perhaps the simplest way to achieve this might be through the ancestral “regulon” strategy employed in bacteria to coordinate biosynthetic pathway enzyme expression [2]. Neurons of a particular NtI would thus express a master regulatory transcription factor that is able to turn on the expression of all genes encoding a particular NtI neurotransmitter identity through its ability to bind a common cis-regulatory element present in all loci. A more complex strategy might involve neuron-subpopulation–specific NtI transcription factors in which different neuron classes use different transcription factors or their combinations to turn on the expression of the same NtI genes. Some recent experiments in the worm *Caenorhabditis elegans* have confirmed the existence of NtI master regulatory transcription factors [3]–[5], raising the question of whether a similar strategy is used in vertebrates. Cho et al. show in vertebrates that such regulatory factors exist but work in a combinatorial manner. The study focuses on two cholinergic neuronal populations: spinal motor neurons (MNs) and forebrain cholinergic neurons (FCNs) [6]. MNs are an ideal starting point for this analysis as much is known about how their genesis is controlled at the transcriptional level [7], [8]. One of the key inducers of MN fate, including their cholinergic NtI, is a hexameric DNA-binding complex containing the LIM-homeodomain transcription factors Lhx3 and Isl1 joined together by the LIM-interactor protein NLI. Such “Isl1-Lhx3 hexamers” are sufficient to induce ectopic MNs exhibiting cholinergic features in the spinal cord and from undifferentiated precursors [9].

The authors first considered the DNA sequences surrounding “cholinergic genes” such as those encoding choline transporters and choline synthesis enzymes. Chromatin immunoprecipitation sequence analysis revealed that the Isl1-Lhx3 hexamer binds conserved regions of these genes, both in embryonic stem (ES) cells and spinal cords. Overexpression of the Isl1-Lhx3 hexamer results in induction of expression of cholinergic genes in the spinal cord, and loss of Isl1 function leads to their down-regulation. Next, using a transcription reporter, the authors showed that the conserved sequences in the cholinergic genes are sufficient for expression in the presence of the Isl1-Lhx3 hexamer and can be further narrowed down to a core response element (HxRE). When multimerized, the HxRE can selectively drive green fluorescent protein (GFP) expression in motor neurons. Next, a developmental time course analysis revealed that FCNs originating from the medial ganglionic eminence express Isl1, NLI, and a distinct LIM-homeodomain (LIM-HD) protein, Lhx8. Notably, deletion of Isl1 from this region results in a depletion of FCNs, reminiscent of the loss of MNs in the spinal cord [10]. Biochemical manipulations next showed that Lhx8 forms a complex with NLI and Isl1 that is analogous to the Isl1-Lhx3 hexamer and that the Isl1-Lhx8 complex binds a specific DNA motif remarkably similar to that recognized by the Isl1-Lhx3 hexamer. This motif is also present in many cholinergic enhancers, and its multimerization is able to drive GFP reporter expression selectively within the embryonic forebrain.

**Figure 1:**
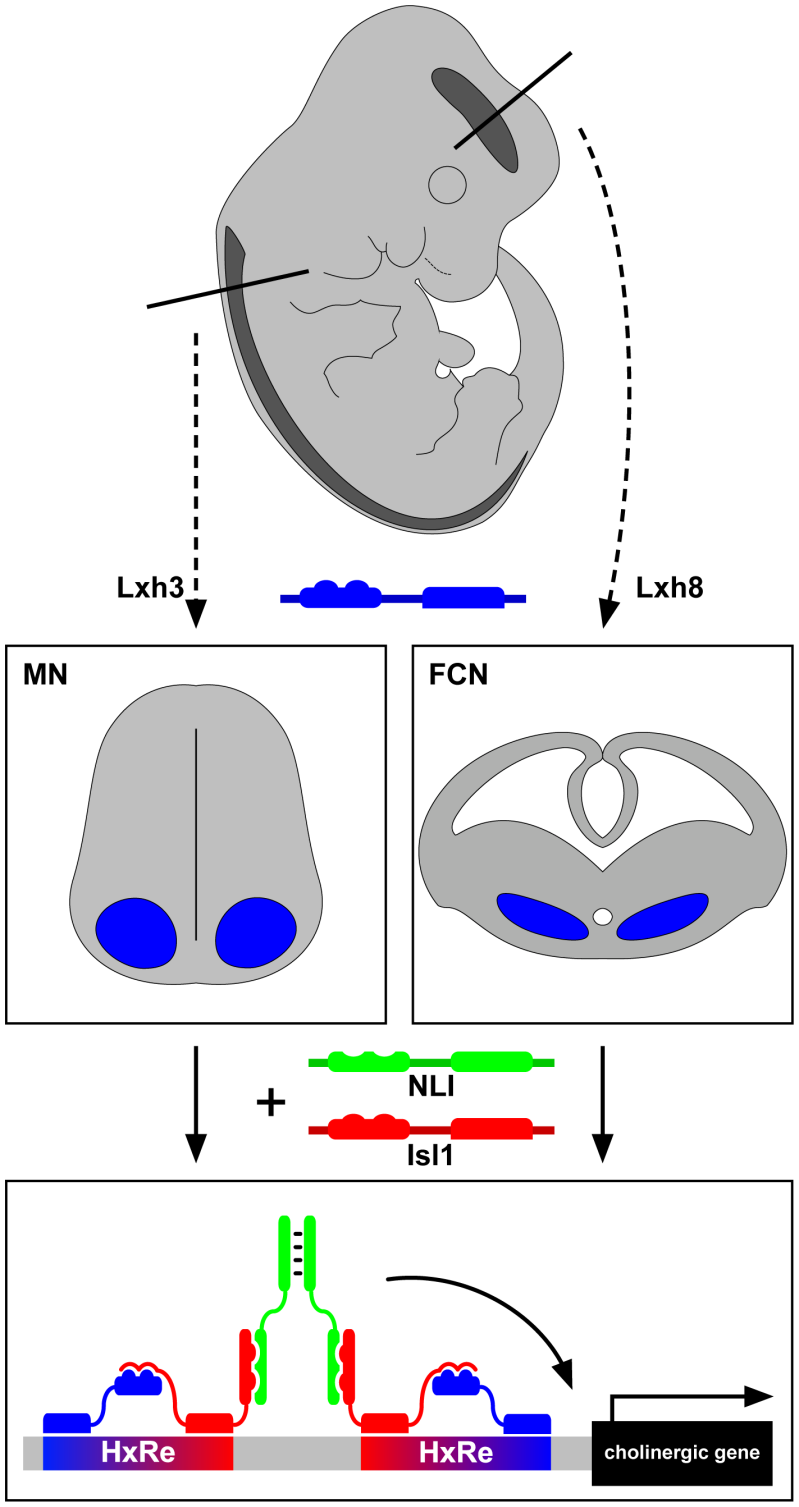
LIM-homeodomain proteins Lhx3 and Lhx8 induce the cholinergic neurotransmitter identity in spinal motor neurons and forebrain cholinergic neurons through Isl1 and NLI hexamer complex transcriptional activity.

Are these hexamers interchangeable? Overexpression experiments argue that only Isl1-Lhx8 hexamer is able to activate cholinergic features in the forebrain, while Isl1-Lhx3 can only induce motor-neuron–specific genes such as Hb9 and Isl2 in the spinal cord, arguing that the cellular context is critical for the function of the Isl1-LhxX hexamers. Extending this conclusion, the production of the Isl1-Lhx8 hexamer in an ES cell line can induce the expression of cholinergic genes but not motor neuron genes. All these data point to the conclusion that Isl1 controls cholinergic NtI in two different regions of the central nervous system through neuron-subtype–specific transcription factor complexes (see Figure 1).

What does it all mean? Clearly, Isl1 is a central player in vertebrate cholinergic NtI whose molecular logic is more complex than that of the simple bacterial regulon. Isl1 works in concert with at least two other neuron-subtype–specific LIM-homeodomain proteins, Lhx1 and Lhx8, which apparently target common enhancer regions near “cholinergic genes.” This is also different from the *C. elegans* strategy in which the UNC-3 transcription factor is sufficient to coregulate all “cholinergic genes” [4]. The most pressing question stemming from this study is the identity of the sequences bound by Isl1-Lhx8 and Isl1-Lhx3. Some preliminary and unpublished observations suggest that these are distinct. One corollary would be that there are other cholinergic neuron-subtype–specific Isl1-Lhx complexes and that each of these has a binding sequence near cholinergic genes. This would make for quite a baroque model of vertebrate cholinergic NtI. Another possibility could be that there are neuron-type–specific Isl1-Lhx hexamers but that these bind to the same DNA sequences. They would only be able to turn on cholinergic genes through the interaction of the Isl1 partner (Lhx, Lhx8, etc) with another cell-type–specific transcription factor binding to nearby sequences.

Arguably, NtI must be tightly regulated so that neurons do not express inappropriate neurotransmitters and adhere (mostly) to Dale's principle that neurons use a single mode of transmission [11]. One scheme could involve active suppression of “competing” NtIs, in which, for example, Isl1-Lhx8 could bind promoters of glutamatergic and GABAergic genes and actively shut them down. This does not appear to be a strategy in *C. elegans*, but vertebrate NtI could be more tortuous [12]. In the broader context, this study provides some attractive models in which to examine the intersection between NtI and other aspects of neuronal identity, such as dendrite morphology or soma location. To what extent are all these linked? Could they really be thought of as separate modules or would a selection of a particular NtI favour some morphological features? This could be regulated either directly at a transcriptional level, in which Isl1-Lhx8, for example, might influence the expression of some FCN “structural” genes, or through homeostatic mechanisms in which the use of a particular neurotransmitter has global consequences on the electrical properties of a neuron. Finally, another important general question is that of the maintenance of neuronal identity, including NtI. Could the same transcriptional complexes that are turning on cholinergy also be required for maintaining it? Could Alzheimer disease and amyotrophic lateral sclerosis, two human pathologies affecting cholinergic neurons, involve a loss of Isl1-Lhx3 and Isl1-Lhx8 function? Some answers to this question will certainly come from a deeper understanding of molecular strategies controlling the intersection of NtI with other aspects of neuronal identity.
